# Empowering young people—the impact of camp experiences on personal resources, well-being, and community building

**DOI:** 10.3389/fpsyg.2024.1348050

**Published:** 2024-02-14

**Authors:** Esther Kirchhoff, Roger Keller, Barbara Blanc

**Affiliations:** Zurich University of Teacher Education, Zürich, Switzerland

**Keywords:** camp activities, caring support, community building, personal resources, positive youth development, social-emotional learning, well-being

## Abstract

**Introduction:**

Personal resources and resources of the sociocultural environment help children and adolescents to successfully cope with challenges in everyday life, which is associated with better individual well-being. SCOUT, the ‘Study on Competence development in OUT-of-school settings’, investigated whether participation in a summer camp enhanced adolescents’ personal resources, well-being, and readiness to contribute to the community.

**Methods:**

The research took place during the Swiss National Jamboree of the Swiss Guide and Scout Movement, a two-week event in the summer of 2022, with a paper-pencil pretest (beginning of the camp – T1) and posttest (end of the camp – T2) survey (*N* = 607, aged 14–17). Confirmatory factor analyses were used to examine whether personal resources, well-being, and readiness to contribute to the community changed over time, and structural equation models were applied to test the direct and indirect effects of caring support from group leaders on the development of these variables.

**Results:**

In less than two weeks, camp participants demonstrated increased empathy, emotional self-control, optimism, and assertiveness. Furthermore, the adolescents reported more positive emotions, higher self-esteem, and stronger readiness to contribute to the community. Group leaders played a crucial role by influencing the positive development of well-being and readiness to contribute to the community both directly and indirectly through the promotion of personal resources.

**Discussion:**

The findings indicate that young people benefit not only from participating in collaborative activities in a stimulating environment, but also from caring support provided by their group leaders.

## 1 Introduction

Growing up is a complex interaction of physical maturation, and the satisfaction of individual needs, and societal expectations (Oerter and Dreher, 2008). Children and adolescents are challenged to deal with their own behaviors, attitudes, and values, to take responsibility, and to live and work together with a diversity of people. Personal resources and resources of the sociocultural environment help them to cope with these developmental tasks (Geldhof et al., 2013; Hurrelmann and Quenzel, 2015; Lohaus and Nussbeck, 2016). Important personal resources are social–emotional skills, positive attitudes, and life expectations, which are also conceptualized as life skills (WHO, 1994, 2003), social and emotional learning (Oberle et al., 2016; Osher et al., 2016), transferable skills, or transformative competencies (OECD, 2019; UNICEF, 2019). A significant resource in the sociocultural context is belonging to a community where people can actively learn, and where they receive support that helps them to shape the environment and their lives in a responsible and productive way (Sameroff, 2010; Lerner, 2017; Lerner et al., 2019; Burkhard et al., 2020).

Equipped with these resources, people are more motivated to learn and use their abilities to work productively, cope effectively with the demands of everyday life, take care of themselves, and contribute to their communities. Consequently, they experience increased well-being, which strengthens physical and mental health and social integration and reduces the risk of health and social problems such as addiction, violence, mental disorders, or unemployment (Lerner, 2017; Taylor et al., 2017; MacArthur et al., 2018; WHO, 2018). The last point, unemployment, is gaining importance as the labor market increasingly expects from employees to have not only technical but also transferable skills (OECD, 2019; Dawson and Harrison, 2023).

Besides family and school, youth organizations are important fields of socialization and learning for children and adolescents (Tomasik et al., 2019). The Positive Youth Development (PYD) approach highlights two essential elements that characterize the services offered by youth organizations: (1) facilitating an active engagement in a variety of leisure activities, including opportunities to take on leadership roles, and (2) creating affective and long-lasting relationships with peers as well as supportive adults working with the young people as mentors or group leaders (Lerner, 2017; Lerner et al., 2019).

Opportunities to actively engage in meaningful and supportive environments have been shown to be important drivers of successful development across the lifespan and have the potential to reduce inequalities in health, especially when the focus is on solidary relationships among members that promote both the individual and collective development (Jang et al., 2014; Oberle et al., 2016; Dibben et al., 2017; Taylor et al., 2017; Immordino-Yang et al., 2019; Lerner et al., 2019; Berger et al., 2020; Dawson and Harrison, 2023).

To summarize, youth organizations following the PYD approach provide opportunities to strengthen the resources of children and adolescents and to build and maintain supportive relationships with peers and adults. In this study, we focused on one youth organization, the Swiss Guide and Scout Movement, and examined the short-term effects of a summer camp on the development of personal resources, well-being, and the readiness to contribute to the community.

### 1.1 Personal resources, well-being, and contribution to community as indicators of PYD

Personal resources help people to cope with challenges in everyday life and they have a positive influence on the individual well-being (Diener and Fujita, 1995; Proctor et al., 2009; Lohaus and Nussbeck, 2016; Taylor et al., 2017). The various conceptualizations of personal resources contain a wide range of interrelated emotional, cognitive, and social skills, positive attitudes and life expectations which lead to increased self-awareness and self-regulation, more informed and responsible decision-making, and abilities and attitudes to build supportive social relationships (Kirchhoff and Keller, 2021). Within the PYD approach, personal resources are described as the “5 Cs” (Lerner et al., 2019): Connection (developing positive relationships that provide a sense of safety and belonging); Competence (developing a positive view of one’s own abilities and skills); Confidence (developing self-confidence, as well as trust in others and confidence in the future); Character (taking responsibility for one’s own actions, and following rules); and Caring (developing compassion and tolerance for others).

In addition, research shows that fostering personal resources in the school setting (Durlak et al., 2011; Taylor et al., 2017; Kirchhoff and Keller, 2021; Mertens et al., 2022) as well as in out-of-school settings (Durlak et al., 2010; Ciocanel et al., 2017; Skeen et al., 2019; Singla et al., 2020) yielded small to moderate effects on a wide range of positive and negative developmental outcomes, such as higher self-esteem, less emotional distress or internalizing problems, more positive social behavior and fewer conduct disorders, as well as higher bonding to school, learning motivation and academic achievement. Children and adolescents with more personal resources are also assumed to behave more carefully in risky or problematic situations (Bonell et al., 2015; Ciocanel et al., 2017; MacArthur et al., 2018; OECD, 2021) and to show a strong motivation to contribute – beyond their own interests – to the development of their families, peers, communities, and ultimately, the civil society (Lerner, 2017; Lerner et al., 2019).

Some of these effects have been shown to unfold more in the longer term, as young people might increasingly benefit from the attitudes and coping skills they have developed. In other words, such learning processes require time and ongoing opportunities to practice the skills and internalize the attitudes (Hodge et al., 2013; Holt et al., 2017; Taylor et al., 2017; Mertens et al., 2022).

Furthermore, a high well-being is also associated with many favorable developmental outcomes, i.e., better academic performance (Bücker et al., 2018; Kaya and Erdem, 2021) as well as enhanced mental and physical health (Proctor et al., 2009; Houben et al., 2015; Taylor et al., 2017; Stifter et al., 2020; WHO, 2020). Well-being often refers to a multidimensional concept that includes both psychological and physical components, e.g., happiness, life satisfaction, positive attitudes, high self-esteem, low worries, and low physical complaints (Hascher et al., 2018). In terms of psychological components, a substantial body of the well-being research focuses on the hedonic dimension of well-being, i.e., on the extent of positive emotions like joy, contentment, and enthusiasm (positive affect, PA), and of negative emotions such as sadness, anger, and anxiety (negative affect, NA) (Watson et al., 1988; Diener et al., 1999; Carver and Scheier, 2009; Houben et al., 2015; OECD, 2021). Other research emphasizes the eudaimonic dimension of well-being, i.e., the evaluation of functioning in life, such as self-esteem and life satisfaction (Ryff and Keyes, 1995; Diener et al., 1999; Ryff, 2014; Houben et al., 2015; Kaya and Erdem, 2021).

Both the affective and evaluative components of well-being influence the focus of attention, thinking and decision-making, as well as action or the readiness to act, respectively (Immordino-Yang and Damasio, 2007; Reis and Gray, 2009). Even slight positive emotions in neutral situations inspire individuals to approach and explore new objects, individuals, or situations. They stimulate the pursuit of personal goals by motivating task-related actions and efforts, and encourage individuals to engage in social activities, build relationships and care about others, and they counterbalance negative emotions (Watson et al., 1988; Fredrickson, 2004; Immordino-Yang and Damasio, 2007; Carver and Scheier, 2009; Proctor et al., 2009; Stifter et al., 2020).

Finally, it is important to note that children’s well-being on average is in the positive range, with a slight decline at the beginning of adolescence (Proctor et al., 2009; Chen et al., 2020; OECD, 2021). Moreover, well-being is influenced to some extent by daily events. In general, positive emotions tend to fluctuate more than negative emotions throughout the day, depending on whether basic needs are satisfied, personal interests can be pursued, or pleasant experiences occur (Watson et al., 1988; Diener et al., 1999).

### 1.2 The role of a supportive environment for PYD

All the approaches mentioned above which aim to strengthen personal resources also emphasize the significance of a supportive environment for the positive development of the individuals and the collective (e.g., Durlak et al., 2010; Lerner et al., 2019). There are some key elements that characterize such an environment. One important aspect is having enough time to build stable relationships (Jang et al., 2014; Ciocanel et al., 2017). Another essential issue is physical and emotional safety, as well as caring support from mentors, or group leaders, for example (Durlak et al., 2011; Immordino-Yang et al., 2019; Lerner et al., 2019; National Academies of Sciences, Engineering, and Medicine, 2019). This includes offering help when needed, showing genuine interest, expressing trust and encouragement, listening to the individual’s concerns as well as providing the opportunity to safely explore and process one’s own needs and ideas, but also offering constructive feedback on undesirable behaviors with clear expression of expectations (Chu et al., 2010; Gariépy et al., 2016; Lerner et al., 2019).

If such a supportive environment can be established, it will not only increase the well-being of children and adolescents, but also empower them to better cope with stress, strengthen their self-esteem, and enhance their self-efficacy and problem-solving skills (Chu et al., 2010; Gariépy et al., 2016). Regarding the role of caring support, Holt et al. (2017) and Cronin and Allen (2018) assume that it can have a positive influence not only directly on well-being, but also indirectly, i.e., through the promotion of personal resources.

### 1.3 Summer camps as opportunities to promote personal resources, well-being, and community building

Youth organizations often follow the principles of PYD. They offer children and adolescents a learning setting from an early age with opportunities to develop self-determined ideas and implement them together. Through regular collaborative activities, participants form strong and long-lasting bonds of solidarity and support to each other. The group members take on specific roles and tasks so that they not only develop their skills but also actively contribute to the group life. As they mature in these youth organizations, some of the young people assume leadership roles. To equip them with the skills needed for effective leadership, they frequently receive guidance and mentorship from more experienced leaders within local or national networks that offer educational programs (Durlak et al., 2010; Ciocanel et al., 2017; Lerner et al., 2019; Berger et al., 2020; Burkhard et al., 2020).

Recent studies have shown that participation in structured out-of-school programs has promising outcomes, particularly in the context of camps (National Academies of Sciences, Engineering, and Medicine, 2019) such as those with immersive nature experiences (Mygind et al., 2019). Camps provide an opportunity to learn and practice skills and to build and maintain supportive relationships with peers and adults. It has been found that camps have positive effects on social skills that foster strong peer relationships, such as empathy, altruism, and perspective-taking, on general self-esteem, self-efficacy, or resilience, and even on academic and cognitive performance (Rose et al., 2018; Whittington and Aspelmeier, 2018; Mygind et al., 2019; Carpio de los Pinos et al., 2020; Gerber et al., 2022). However, there are also inconsistent findings, e.g., regarding problem solving, aggression, and internalizing problems such as fear, personal distress, depression, and mood (Rose et al., 2018; Mygind et al., 2019; National Academies of Sciences, Engineering, and Medicine, 2019; Carpio de los Pinos et al., 2020). In general, effects were stronger when children and adolescents were familiar with the specific living conditions in the camp and with the people participating (Gerber et al., 2022) and when they felt physically and emotionally safe (Garst et al., 2016; Whittington et al., 2017; Richmond et al., 2019; Garst and Whittington, 2020).

### 1.4 Aim of the study

This study focused on the Swiss Guide and Scout Movement, the largest youth organization in Switzerland with more than 51,000 members. With its organizational structure and goals, it fulfills the two pillars of PYD programs mentioned above, i.e., (1) facilitating an active engagement in a variety of leisure activities, including opportunities to take on leadership roles, and (2) creating affective and long-lasting relationships with peers as well as supportive young adults working as group leaders (Pfadibewegung Schweiz, 2008; Kalcsics et al., 2011; Lerner et al., 2019). Scouting Switzerland offers unique group experiences in nature during Saturday activities, training courses or camps, including young people regardless of their origin and cultural or religious background.

In line with research showing positive effects of out-of-school settings on youth development (e.g., Durlak et al., 2010; Jang et al., 2014; Ciocanel et al., 2017; Lerner et al., 2019; Dawson and Harrison, 2023), SCOUT, the ‘Study on Competence development in OUT-of-school settings’ investigated the benefits of the Swiss National Jamboree, a two-week summer camp organized by the Swiss Guide and Scout Movement. The aim of the study was to examine whether participation in the camp led to more personal resources, better well-being, and an increased readiness to contribute to the community. Furthermore, as suggested by Cronin and Allen (2018) and Holt et al. (2017), the study explored whether caring support received from group leaders, as an integral component of a supportive environment, facilitated the adolescents’ positive development.

We hypothesized the following:

*H1*: Camp participation leads to a significant increase in nine personal resources from the beginning of the camp (T1) until shortly before the end of the camp (T2).

*H2*: Camp participation leads to a significant increase in three indicators of well-being from T1 to T2.

*H3*: Camp participation leads to a significant increase in the readiness to contribute to the community from T1 to T2.

*H4*: Received caring support from group leaders has both a direct moderating effect on the development of personal resources, well-being, and the readiness to contribute to the community, and a mediating effect on well-being and contribution to the community through the promotion of personal resources.

In the selected setting, the assumed changes over time can be largely attributed to the camp activities, as there are no significant influences from family, school, or other leisure activities during this time. If the study can demonstrate short-term effects, it would provide evidence that camps serve as a meaningful out-of-school learning environment, promoting personal resources, well-being, and community building, which in turn leads to a positive and healthy development in children and youth.

## 2 Materials and methods

The study was conducted during the summer camp (Swiss National Jamboree) in the Goms Valley of Switzerland from July 23 to August 6, 2022. Around 30,000 scouts aged 6 to 17 from different language regions of Switzerland and nine other countries took part in the camp, with hikes, off-road games, campfires, handicraft activities, water sports and many other activities.

The focus in this study was on the “Venture Scouts.” These are 14- to 17-year-old adolescents who organize their own activities under the supervision of their group leaders. At the end of their time as Venture Scouts, they have the opportunity to gain their first experiences as group leaders.

### 2.1 Procedures and participants

The Ventures Scouts were selected for reasons of feasibility. In Switzerland, adolescents aged 14 years and older are legally permitted to sign themselves the consent form for study participation. Younger participants could not be included because parents could not be contacted in advance for written consent.

#### 2.1.1 Recruitment

**Figure 1:**
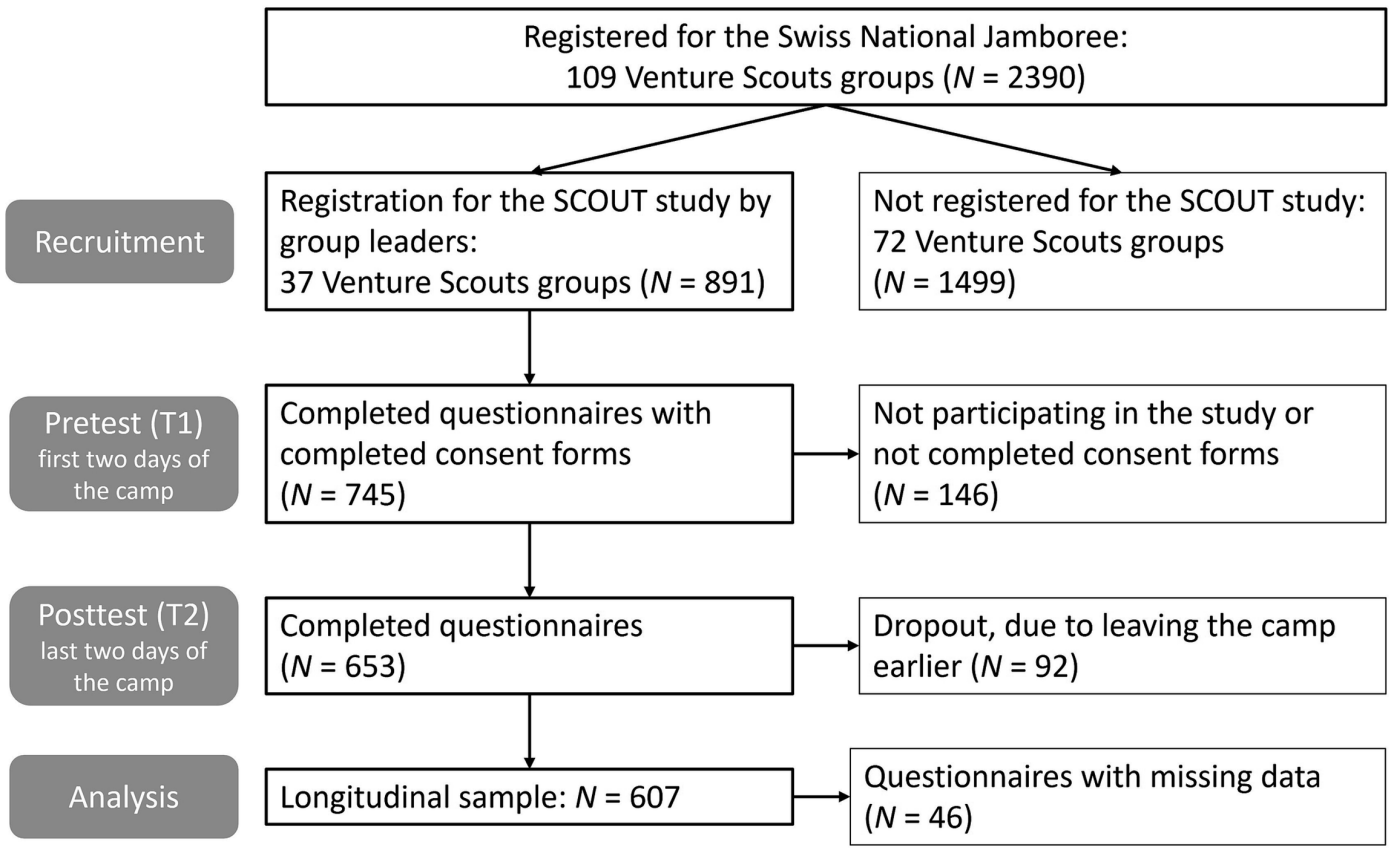
Flowchart of sample recruitment, data collection, and data analysis.

A total of 109 groups, comprising *N* = 2,390 Venture Scouts, registered for the Swiss National Jamboree (see Figure 1). In collaboration with the administrative office of the Swiss Guide and Scout movement and after approval by the regional Scout associations, the research team contacted in advance the leaders of the Venture Scouts groups by e-mail, sending them information (time points, locations, and duration of the survey) and arguments for participating in the study (to scientifically demonstrate which resources young people acquire in Scouting). In addition, the research team provided an access link for registration in order to plan the data collection. As Scouts groups in Switzerland are legal entities, their group leaders had the autonomy to decide whether their group took part in the study or not. 37 Venture Scout groups with *N* = 891 adolescents agreed to participate in the study.

#### 2.1.2 Data collection

The Venture Scouts were surveyed twice during the camp using a paper-pencil questionnaire. The first measurement (pretest, T1) took place in the first two days of the camp, while the second measurement (posttest, T2) was conducted after approximately 12 days, i.e., in the last two days of the camp. The survey was conducted completely anonymously at the camp site of the groups by the research team. Filling out the questionnaires took 20 to 30 min. As an incentive, the participants received a pin featuring the camp’s motto (“Learned is learned”) after T2, while the group leaders obtained a packed lunch for the organizational effort.

#### 2.1.3 Data preparation

The questionnaires were scanned using the survey software evasys (evasys GmbH, 2022), and the data were transferred to an electronic data set. At T1, a total of *N* = 745 participants aged 14 to 17 with fully completed consent forms filled out the questionnaire, and at T2, *N* = 653 (see Figure 1). The smaller sample size at T2 was mainly because some adolescents started their vocational apprenticeship at the beginning of August 2022 and therefore had to leave the camp earlier. The two questionnaires (T1 and T2) were merged by means of a personal camp code which the participants had to indicate on the questionnaires. The participants’ codes and group information were completely anonymized.

The total sample for the longitudinal analyses was *N* = 607, as some participants had missing responses, such as not paying attention to the reverse sides of the six-page questionnaires.

#### 2.1.4 Participants

Table 1 provides an overview of the longitudinal sample. The average age of the adolescents was *M* = 15.46 (*SD* = 0.96), with the majority of adolescents aged 15 and 16. The gender distribution was balanced (female: 51.2%, male: 48.8%), and the distribution across the three language regions corresponded to the situation in Switzerland.

**Table 1:** Sample description (*N* = 607).

Variable		*n*	%
Gender	Female	311	51.2
	Male	296	48.8
Age	14 years	108	17.8
	15 years	205	33.8
	16 years	199	32.8
	17 years	95	15.6
Language region	German	334	55.0
	French	161	26.5
	Italian	112	18.5

### 2.2 Measures

The questionnaires were developed in German, with items from English-language scales being translated into German. In a second step, the German versions of the questionnaires were translated into French and Italian (if a corresponding language version of the measures were not yet available). The translations were checked by native-speaking researchers and members of the Swiss Guide and Scout Movement.

All scales were assessed using a seven-point Likert scale, ranging from 1 (not at all true) to 7 (completely true). For most questions (unless otherwise noted in the description below) the instruction was “Please answer the following questions, even if you are not completely sure.” Except for caring support from group leaders received at camp, all scales were collected on both measurements, i.e., at the beginning and end of camp.

#### 2.2.1 Personal resources

Nine concepts were chosen aligning the Core Values of Scouting in Switzerland (Kalcsics et al., 2011), the “5 Cs” of PYD (Lerner, 2017; Lerner et al., 2019), and the Life Skills approach (WHO, 1994, 2003; Kirchhoff and Keller, 2021). Three scales measured *interpersonal skills* that ensure that not only the needs and conditions of others, but also one’s own needs and conditions were met in social interactions (Groeben et al., 2011; OECD, 2021)—a prerequisite for positive and close relationships: *Empathy* was measured by a six-item scale from the German *Fragebogen zu Ressourcen im Kindes-und Jugendalter* [Questionnaire on resources in childhood and adolescence] (FRKJ 8–16) by Lohaus and Nussbeck (2016), including affective and cognitive aspects (Cronbach’s *α*(T1) = 0.850; *α*(T2) = 0.866; item example: “I can relate well to how others feel”). To measure *Caring*, four of the eight items of a scale of the Positive Youth Development (PYD) Inventory developed by Arnold et al. (2012) were taken (Cronbach’s *α*(T1) = 0.712; *α*(T2) = 0.740; item example: “I can be counted on to help if someone needs me”). *Assertiveness* was measured by six items taken from the Perceived Social Efficacy and the Perceived Self-regulatory scales, both developed by Basili et al. (2020), and a seventh item from the Life Skills Training Questionnaire developed by the National Health Promotion Associates (2019) (Cronbach’s *α*(T1) = 0.854; *α*(T2) = 0.863; item example: “Are you able to … stand up for yourself when you feel you have been treated unfairly?”).

One scale measured the more *cognitive and methodical skills*: *Problem-solving and decision-making* were adapted from five of nine items which Mincemoyer and Perkins (2005) developed for a Youth Life Skills Evaluation System (Cronbach’s *α*(T1) = 0.800; *α*(T2) = 0.863; item example: “I’m trying to figure out exactly what the problem is”).

Two scales represented the *self-regulation and self-management skills*: *Effort* was measured by six of the seven items developed by Anderson-Butcher et al. (2016a) (Cronbach’s *α*(T1) = 0.820; *α*(T2) = 0.854; item example: “Even when things get difficult, I do my best”). *Emotional self-control* was measured by seven items, adapted from a scale with eight items developed also by Anderson-Butcher et al. (2016b) (Cronbach’s *α*(T1) = 0.822; *α*(T2) = 0.854; item example: “I can calm down when something upsets me”).

Three scales measured *positive attitudes and life expectations*, i.e., personal resources that energize the motivation to face the demands of life (OECD, 2021; Dawson and Harrison, 2023). They were all taken from the already mentioned FRKJ 8–16 (Lohaus and Nussbeck, 2016), each one measured by six items: *Optimism* (Cronbach’s *α*(T1) = 0.859; *α*(T2) = 0.885; item example: “I look forward to my future with confidence”), *Self-efficacy* (Cronbach’s *α*(T1) = 0.887; *α*(T2) = 0.908; item example: “When I set myself a goal, I achieve it”), and *Sense of coherence* (Cronbach’s *α*(T1) = 0.807; *α*(T2) = 0.838; item example: “I can affect my life”).

#### 2.2.2 Well-being

To measure the affective as well as the evaluative dimension of well-being, three indicators were assessed: positive and negative emotions, and self-esteem. To measure positive and negative emotions, the two dimensions of the PANAS were used: positive affect (PA) and negative affect (NA), each comprising ten items. The participants were asked to indicate how they felt the preceding two weeks (thus, for T2, spanning the duration of the camp). For the German questionnaire, the items from Krohne et al. (1996) were used. The French items were adapted from an online version of the PANAS provided by the Institut Français d’EMDR (2022), and from Gaudreau et al. (2006). The Italian version was adapted from Terracciano et al. (2003). For the analyses, one item of the PA dimension, i.e., “alert,” was removed due to not meeting goodness-of-fit criteria in factor and reliability analyses, especially at T2. It can be assumed that participants interpreted this item less as an emotional and more as a physical state, as they experienced probably limited sleep during the camp. Thus, PA included nine items referring to an enthusiastic, active, and interested state (Cronbach’s *α*(T1) = 0.814; *α*(T2) = 0.842). NA, with its ten items, referred to a variety of feelings of being nervous, afraid, or upset (Cronbach *α*(T1) = 0.849; *α*(T2) = 0.870). *Self-esteem* was measured by a six-item scale taken from the mentioned FRKJ 8–16 (Lohaus and Nussbeck, 2016). For the analyses, an inversely formulated item had to be excluded to improve scale consistency (for the remaining five-item scale: Cronbach’s *α*(T1) = 0.916; *α*(T2) = 0.931; item example: “I feel good thinking about myself”).

#### 2.2.3 Readiness to contribute to the community

This indicator for community building was measured by four items taken from a seven-item scale from the PYD Inventory developed by Arnold et al. (2012) (Cronbach’s *α*(T1) = 0.705; *α*(T2) = 0.760; item example: “I take an active role in my community”).

#### 2.2.4 Received caring support by group leaders

At T2, the participants rated on four items the caring support received from their group leaders during the camp. The scale was adopted from the Health Behavior in School-aged Children (HBSC) study 2018 (Delgrande Jordan et al., 2019), because it has already been tested in several language versions and includes various aspects of caring support. The items were reformulated for group leaders (Cronbach’s *α*(T2) = 0.882; item example: “I had leaders with whom I could share my joys and sorrows”).

### 2.3 Data analysis

After checking the quality for each scale and measurement time point separately (in SPSS, version 28.0.1.0, and Mplus, version 8.6), the items were grouped into three parcels per scale and measurement time point. A balanced parceling strategy was applied (Little et al., 2013). All further structural equation models (SEM) were analyzed in Mplus. Robust Maximum Likelihood (MLR) was used to estimate the models, with Full Information Maximum Likelihood (FIML) to handle missing data. The common criteria found in literature were used to interpret the model fits: *χ^2^*/*df* ratio ≤ 2.5; Root Mean Square of Approximation (RMSEA) ≤ 0.06; Comparative Fit Index (CFI) ≥ 0.95, and Standardized Root Mean Residual (SRMR) ≤ 0.08 (Weiber and Mühlhaus, 2014; Kleinke et al., 2017; Geiser, 2020). In addition, to compare nested models, e.g., in analyses of measurement invariance or when models with a latent interaction term have been tested, the loglikelihood (LL) values and the information criteria Akaike Information Criterion (AIC), as well as the sample-size adjusted Bayesian Information Criterion (sBIC) were considered, too. Regarding LL, the Sattora-Bentler *χ^2^* difference test based on the LL values and scaling correction factors obtained with the MLR estimator was used (Satorra and Bentler, 2010; Muthen & Muthen, 2023). In addition, for the AIC, we followed a recommendation of Hallquist and Wright (2014) to interpret two models as substantially different, if the differences of the AIC values were larger than ± 10.

To test hypotheses 1 to 3, confirmatory factor analyses (CFA) over time were estimated within each scale separately, including testing the measurement invariance over time. At strong (scalar) measurement invariance the factor means at T1 were set to 0 and the factor means at T2 were freely estimated, thus allowing to determine if changes over time were significantly different from 0. However, as the model fits of these CFAs across time were clearly insufficient (results not shown in detail), indicator-specific residual factors (IS_i_ factors) (Geiser, 2020) were included in all further analyses. The IS_i_ factors were allowed to correlate with each other, but not with the latent factors of the models. This supplement enhanced the model fits of the CFAs over time to a partly almost perfect level (parameter estimations of the IS_i_ factors are not reported further).

To test hypothesis 4, all latent variables proven to change significantly between T1 and T2 (see hypotheses 1 to 3) were first examined in separate SEMs. We controlled the T2 levels of the variables for their T1 levels (autoregressive paths), thus enabling to interpret the T2 values as intraindividual changes over time (Kleinke et al., 2017). However, due to better model fits (details not shown), weak (metric) measurement invariance was used in further analyses. Then we regressed the T2 levels of the variables on received caring support from group leaders at T2, thus examining the extent to which received caring support moderated the intraindividual changes in the variables. Correlations between the latent variables at T1 and caring support at T2 were set to zero. Finally, to test the complete moderation effect of caring support, a latent interaction term was included, by multiplying the variable level at T1 and the level of caring support at T2 (Kleinke et al., 2017). As this latter method belongs to mixture analysis, only the LL and the information criteria were available in Mplus to compare models. Significant moderation effects were analyzed graphically, with a method shown in Kleinke et al. (2017).

After determining the significance of the moderation effects for each scale under examination, we brought all latent variables together in an overall model to test the mediation hypothesis. First, the T2 level of each latent variable was again (1) controlled for its T1 levels (autoregressive paths), and (2) regressed on caring support from group leaders and/or the interaction term if these moderation effects have been shown to be significant in the step before. Second, we included the mediating paths from the T2 personal resource variables to the T2 indicators of well-being and readiness to contribute to the community. To compare these two models, we descriptively interpreted the model fits, the autoregressive paths, the correlations as well as the direct, indirect, and total effects of caring support on the indicators of well-being and on readiness to contribute to the community.

## 3 Results

### 3.1 Developments during the summer camp

The hypothesized positive developments over time were partially confirmed. Within less than two weeks, significant improvements (*p* < 0.01 to *p* < 0.001, see Table 2) were observed in empathy, assertiveness, emotional self-control, and optimism as personal resources (H1). PA, and self-esteem, both serving as indicators of well-being (H2), and readiness to contribute to the community (H3) also increased significantly. PA showed the largest increase among all variables. There were no significant changes in caring, problem-solving/decision-making, effort, self-efficacy, sense of coherence, and NA.

**Table 2:** CFAs over time, for each variable separately: changes in factor means between T1 and T2, unstandardized factor means and standard deviations for T1 and T2, correlations between factor means of T1 and T2, and model fits (*N* = 607).

Variable	Change at T2^a^	T1	T2	*r*_T1-T2_^a^	Model fits^a^				
		*M* (*SD*) ^b^	*M* (*SD*) ^b^		*χ^2^* (*df* = 7)	*χ^2^*/*df*	RMSEA	CFI	SRMR
Empathy	0.095 **	5.05 (1.08)	5.15 (1.03)	0.751***	4.261 ^ns^	0.609	0.000 ^ns^	1	0.022
Caring	0.020 ^ns^	5.63 (0.88)	5.61 (0.88)	0.735***	2.815 ^ns^	0.402	0.000 ^ns^	1	0.016
Assertiveness	0.100 **	5.63 (1.04)	5.74 (0.99)	0.757***	9.527 ^ns^	1.361	0.024 ^ns^	0.998	0.020
Problem-solving	0.055 ^ns^	5.42 (0.85)	5.44 (0.86)	0.690***	4.545 ^ns^	0.649	0.000 ^ns^	1	0.014
Effort	0.021 ^ns^	5.26 (0.88)	5.28 (0.88)	0.750***	6.509 ^ns^	0.930	0.000 ^ns^	1	0.013
Emotional self-control	0.104 **	4.92 (1.02)	4.97 (1.03)	0.712***	10.159 ^ns^	1.451	0.027 ^ns^	0.998	0.026
Optimism	0.092 **	5.09 (1.13)	5.15 (1.15)	0.806***	4.942 ^ns^	0.706	0.000 ^ns^	1	0.001
Self-efficacy	0.017 ^ns^	5.25 (1.03)	5.23 (1.05)	0.788***	5.728 ^ns^	0.818	0.000 ^ns^	1	0.011
Sense of coherence	0.008 ^ns^	5.44 (0.85)	5.45 (0.86)	0.754***	4.266 ^ns^	0.609	0.000 ^ns^	1	0.020
Positive affect	0.496 ***	4.66 (1.15)	5.01 (1.13)	0.524***	28.454***	4.065	0.071 ^ns^	0.985	0.054
Negative affect	0.053 ^ns^	2.82 (1.10)	2.68 (1.07)	0.630***	38.662***	5.517	0.086 *	0.982	0.025
Self-esteem	0.122 ***	4.94 (1.29)	5.10 (1.25)	0.825***	30.042***	4.292	0.074 ^ns^	0.991	0.026
Contribution	0.124 ***	5.13 (1.09)	5.25 (1.09)	0.688***	7.513 ^ns^	1.073	0.011 ^ns^	0.999	0.020

All values estimated in separate models within each variable. T1, first measurement point at beginning of the camp; T2, second measurement point at the end of the camp; M, mean; SD, standard deviation; Likert scale ranging from 1 (not at all true) to 7 (completely true). Factor loadings not shown in detail, all loadings > 0.5. LL and information criteria model fits are not shown, but mostly confirmed the direction of findings. ^a^Values taken from models with strong measurement invariance. ^b^Values taken from models with weak measurement invariance. RMSEA: the significance indices refer to the probability of the value being ≤ 0.05. ***p ≤ 0.001, **p ≤ 0.01, *p ≤ 0.05, ns, not significant.

As shown in Table 2, the unstandardized factor mean values at T1 of all personal resources showed already high levels at the beginning of the camp, whereas PA, self-esteem, and readiness to contribute to the community showed middle to high levels at the beginning of camp, and NA was low. The bivariate correlations over time were very high for all personal resource variables as well as for self-esteem and readiness to contribute to the community, pointing to a strong ranking stability. The corresponding correlations for PA and NA were slightly lower, especially for PA, indicating more variation in intraindividual changes over time.

### 3.2 Caring support from group leaders and the mediating effect of personal resources

On average, received caring support from group leaders during the camp was rated as relatively high (*M* = 5.60, *SD* = 1.18) and it explained significantly the intraindividual changes between T1 und T2 (H4, see Table 3). For some variables, the inclusion of the variable of caring support yielded even a higher explained variance of the intraindividual changes.

**Table 3:** Changes in factor means between T1 and T2, autoregression (T2 on T1, standardized *β*s), and moderation effects of received caring support from group leaders on these changes (standardized *β*s for main effect and interaction effect of caring support*T1 level) (*N* = 607).

Variable		Change between T1 and T2	Predicting change at T2			Model fits					
			Autoregr. (all *p* < 0.001)	Car. Supp. (all *p* < 0.001)	Interact.	*R^2^* (all *p* < 0.001)	*χ^2^*	*χ^2^*/*df*	RMSEA	CFI	SRMR
Emotional self-control	M1	0.105**	0.704	--	--	0.496	5.132 ^ns^	1.026	0.007 ^ns^	1	0.030
	M2	0.107**	0.686	0.207	--	0.513	24.247 ^ns^	1.102	0.013 ^ns^	0.999	0.067
	M3	0.107**	0.686	0.207	0.000 ^ns^	0.513	LL_M3_ = LL_M2_	AIC_M3_ = AIC_M2_	--	--	--
Assertiveness	M1	0.099**	0.756	--	--	0.572	4.886 ^ns^	0.977	0.000 ^ns^	1	0.020
	M2	0.101**	0.743	0.143	--	0.573	46.251**	2.102	0.043 ^ns^	0.990	0.088
	M3	0.111**	0.735	0.151	−0.054 ^ns^	0.566	LL_M3_ = LL_M2_	AIC_M3_ = AIC_M2_	--	--	--
Empathy	M1	0.096**	0.745	--	--	0.555	3.942 ^ns^	0.788	0.000 ^ns^	1	0.040
	M2	0.097**	0.737	0.169	--	0.571	37.141*	1.688	0.034 ^ns^	0.994	0.051
	M3	0.106**	0.745	0.175	−0.115**	0.599	LL_M3_ < ** LL_M2_	AIC_M3_ < ^t^ AIC_M2_	--	--	--
Optimism	M1	0.097**	0.792	--	--	0.627	8.004 ^ns^	1.601	0.031 ^ns^	0.998	0.073
	M2	0.099**	0.775	0.169	--	0.629	65.971***	2.999	0.057 ^ns^	0.985	0.117
	M3	0.108**	0.774	0.168	−0.042 ^ns^	0.629	LL_M3_ = LL_M2_	AIC_M3_ = AIC_M2_	--	--	--
Positive affect	M1	0.489***	0.516	--	--	0.266	7.002 ^ns^	1.400	0.026 ^ns^	0.999	0.027
	M2	0.508***	0.467	0.288	--	0.301	75.373***	3.426	0.063 ^ns^	0.977	0.108
	M3	0.493***	0.466	0.292	0.063 ^ns^	0.306	LL_M3_ = LL_M2_	AIC_M3_ = AIC_M2_	--	--	--
Self-esteem	M1	0.131***	0.794	--	--	0.631	47.381***	9.476	0.118***	0.984	0.174
	M2	0.133***	0.782	0.139	--	0.631	113.666***	5.167	0.083***	0.975	0.174
	M3	0.138***	0.792	0.136	−0.020 ^ns^	0.631	LL_M3_ = LL_M2_	AIC_M3_ = AIC_M2_	--	--	--
Contribution	M1	0.127**	0.680	--	--	0.462	4.096 ^ns^	0.819	0.000 ^ns^	1	0.016
	M2	0.134**	0.672	0.182	--	0.484	59.631***	2.711	0.053 ^ns^	0.978	0.079
	M3	0.132**	0.672	0.182	0.010 ^ns^	0.485	LL_M3_ = LL_M2_	AIC_M3_ = AIC_M2_	--	--	--

Values taken from models with weak measurement invariance (MI) over time, except for change values between T1 and T2: taken from models with strong MI. All factor loadings > 0.5. M1 = model with autoregression only (autoregr., indicating the ranking stability), 22 free parameters, df(χ^2^) = 5. M2 = model with autoregression and main effect of caring support (Car. Supp.), 32 free parameters, df(χ^2^) = 22. M3 = model with autoregression, main effect of caring support and interaction effect (interact. = T1 level of the variable * caring support), 33 free parameters, *χ*^2^-based model fits not available. RMSEA: the significance indices refer to the probability of the value being ≤ 0.05. LL_M3_ = Loglikelihood value of M3, LL_M2_ = Loglikelihood value of M2, values not shown; significance of LL differences tested using the Satorra-Bentler Test. AIC_M3_ = AIC of M3, AIC_M2_ = AIC of M2, values not shown; ^t^substantially better AIC for M3, compared to M2, according to a recommendation in Hallquist and Wright (2014). ***p ≤ 0.001, **p ≤ 0.01, *p ≤ 0.05, ns, not significant.

The graphical analyses (see Supplementary material) indicated that the higher the received caring support from group leaders as well as the baseline values of the variables at T1, the more favorable the developments were. Most participants benefitted from received caring support, and when a variable’s score at T1 was below average (−1 *SD* from average), caring support influenced whether participants reported increases or decreases in the variable over time. However, for PA with its highest average change between T1 and T2, there were almost no negative changes over time, even when caring support, or PA at T1, respectively, were below average.

Furthermore, the interaction term between received caring support and the T1 level of the variable was found to be significant only for empathy (see Table 3). The graphical analysis showed that for participants with highest initial levels at T1, empathy increased similarly over time, regardless of the level of received caring support.

**Figure 2:**
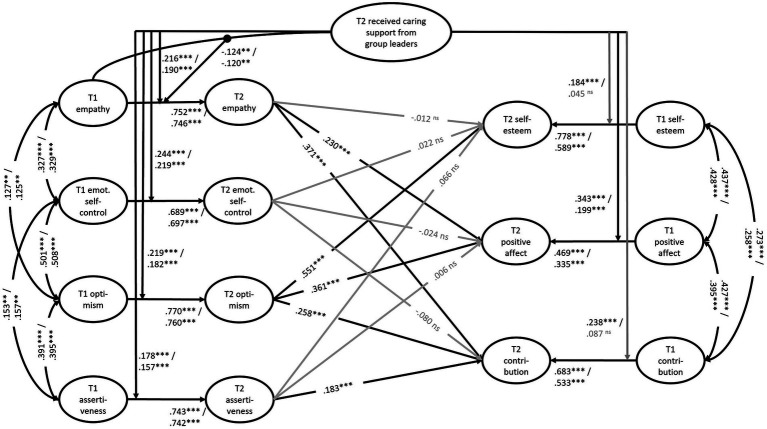
Direct and indirect effects of received caring support from group leaders on changes in well-being and readiness to contribute to the community at T2, with mediation effects via changes in personal resources at T2 (*N* = 607). Legend: Single sided arrows are standardized *β*s; double sided arrows are standardized correlations. Black arrows represent significant values; gray arrows represent insignificant values. If two values are given, they represent estimations from the model without mediation/with mediation effects. If one value is given, it represents the estimation from the model with mediation effects. The filled circle represents the latent interaction term of empathy at T1 multiplied with caring support. Factor indicators are not shown, all factor loadings >0.5. For model fits: see Table 4. ***p ≤ 0.001, **p ≤ 0.01, *p ≤ 0.05, ns = not significant.

Finally, Table 4 and Figure 2 show the results of the overall models to test the mediation part of the hypothesis (H4). The first overall model *without* mediations confirmed the moderating role of received caring support from group leaders on intraindividual changes over time. It should be noted that there were small to medium significant correlations between the personal resource variables at T1, as well as between the indicators of well-being and readiness to contribute to the community at T1, indicating an overlap in the content of the constructs investigated (see Figure 2).

**Table 4:** Explained variances as well as direct and indirect effects of received caring support from group leaders on changes in well-being and readiness to contribute to the community at T2, with mediation effects via changes in personal resources at T2 (*N* = 607).

Model	Changes in self-esteem		Changes in positive affect		Changes in readiness to contribute to the community	
	Without mediation	With mediation	Without mediation	With mediation	Without mediation	With mediation
*R^2^* Outcome T2	63.9%	+ 5.3%	33.7%	+ 5.2%	52.4%	+ 5.8%
Autoregression	0.778***	0.589***	0.469***	0.335***	0.683***	0.533***
Effects of caring support						
Total	--	*0.160*	*--*	*0.276*	*--*	*0.171*
Direct	0.184***	.045^ns^	0.343***	0.199***	0.238***	.087^ns^
Total indirect	--	*0.115*	*--*	*0.077*	*--*	*0.084*
Specific indirect effects via changes in						
Empathy	--	*−0.001*	*--*	*0.016*	*--*	*0.026*
Main effect		(0.190*** x −0.012^ns^)		(0.190*** x 0.230***)		(0.190*** x 0.371***) +
Interaction effect		(−0.120** x −0.12^ns^)		(−0.120** x 0.230***)		(−0.120** x 0.371***)
Emotional self-control (only main effect)	--	*0.005*	*--*	*−0.005*	*--*	*−0.018*
		(0.219*** x 0.022^ns^)		(0.219*** x −0.024^ns^)		(0.219*** x −0.080^ns^)
Optimism (only main effect)	--	*0.100*	*--*	*0.066*	*--*	*0.047*
		(0.182*** x 0.551***)		(0.182*** x 0.361***)		(0.182*** x 0.258***)
Assertiveness (only main effect)	--	*0.010*	*--*	*0.001*	*--*	*0.029*
		(0.157*** x 0.066^ns^)		(0.157*** x 0.006^ns^)		(0.157*** x 0.183***)

Values taken from models with weak measurement invariance over time for all variables. All factor loadings > 0.5. Model fits for the model without mediation: LL = 34,564, AIC = 69,489, sBIC = 69,711; for the model with mediation: LL = 34,349, indicating a significant lower value according to the Satorra-Bentler test; AIC = 69,083, indicating a substantially lower value according to a recommendation in Hallquist and Wright (2014), sBIC = 69,320. Indirect effects: the values in brackets are the respective βs of the mediation paths (see also Figure 2). See Figure 2, too, for the autoregressive paths of the personal resource variables, and for the direct effects of caring support on the personal resources for the model without mediation. Specific indirect, total indirect and total effects of caring support on the variables (all in italics) were calculated by hand, without calculating significances. R^2^ for the personal resource variables at T2, from the models without/with mediation effects (all *p*’s < 0.001): empathy: 0.628/0.607; emotional self-control: 0.534/0.534; optimism: 0.641/0.610; assertiveness.584/0.575. ***p ≤ 0.001, **p ≤ 0.01, *p ≤ 0.05, ns, not significant.

Including the *mediating paths* resulted in an improved model fit (see details in the legend of Table 4), as the effect of caring support from group leaders on changes in PA, self-esteem, and readiness to contribute to the community was mainly mediated through the participants’ personal resources (see Table 4; Figure 2). The effect of caring support on changes in *self-esteem* was fully mediated by its effect on optimism. This resulted in a larger total indirect effect compared to the observed direct effect of caring support on changes in self-esteem. The model with mediation paths increased the explained variance of intraindividual changes in self-esteem by approximately +5.3%.

The influence of caring support on changes in the *readiness to contribute to the community* was fully mediated by its effect on participants’ empathy, optimism, and assertiveness. The total indirect effects from caring support on contribution via these personal resources, however, were of the same size as its (now non-significant) direct path. Nevertheless, the model with mediation paths increased the explained variance of intraindividual changes in the readiness to contribute to the community by approximately +5.8%.

In terms of changes in *PA*, the direct effect of received caring support remained significant and thus was only partly mediated by personal resources, i.e., by optimism and empathy. However, the total indirect effect of caring support on PA via the personal resources was of clearly smaller size than its still significant direct effect. The model with mediation paths increased the explained variance of intraindividual changes in PA by approximately +5.2%.

Emotional self-control was the only personal resource with no mediating effect on the indicators of well-being and on readiness to contribute to the community.

## 4 Discussion

Children and adolescents have to deal with their own behaviors, attitudes and values, take responsibility and live and work together with a diversity of people. Literature shows that personal resources and resources of the sociocultural environment help them to cope with these developmental tasks. As a result, they experience increased well-being, and they are more motivated to actively contribute to the community. This strengthens their mental health and social integration and reduces the risk of health and social problems (Hurrelmann and Quenzel, 2015; Taylor et al., 2017; WHO, 2018; Lerner et al., 2019; Dawson and Harrison, 2023).

The SCOUT study investigated among *N* = 607 adolescents aged 14 to 17 whether the participation in the two-week Swiss National Jamboree of the Swiss Guide and Scout Movement in the summer of 2022 led to more personal resources, better well-being, and an increased readiness to contribute to the community. In addition, it was examined whether caring support received from group leaders, as an important aspect of a supportive environment, was related to such positive development.

Results showed that four personal resources, namely empathy, assertiveness, emotional self-control, and an optimistic view of life and the future, increased significantly within a short period of less than two weeks. Participants also reported higher levels of positive emotions (PA) and self-esteem, along with stable low levels of negative emotions (NA); moreover, their readiness to contribute to the community was increased at the end of the camp.

These results are mostly in line with the literature (Rose et al., 2018; Whittington and Aspelmeier, 2018; Mygind et al., 2019; Carpio de los Pinos et al., 2020; Gerber et al., 2022), particularly with regard to changes in empathy and self-esteem. The camp provided opportunities to strengthen cohesion within the group and to interact with a variety of other young people. These experiences seemed to deepen the participants’ understanding and appreciation not only for others but also for themselves.

In addition, the increased assertiveness refers to a strengthened self-efficacy in social situations that also encompasses resistance against social pressure. Participants may have experienced in the camp that their opinions were acknowledged and that their needs and ideas were respected. This finding is important as self-efficacy in social situations nourishes self-esteem (Mertens et al., 2022), and buffers against emotional distress and depression (Bandura et al., 1999; Groeben et al., 2011).

At the same time, emotional self-control might have been fostered by the generally positive and caring atmosphere within the camp. As emotional self-control has high significance for the regulation of collective life (Mertens et al., 2022), this positive development also is worth noting. Similarly, the increased optimism could be an indication of the positive experiences gained during camp life. The team activities may have fostered an understanding that problems can be solved through collective efforts.

However, the level of other personal resources remained stable throughout the camp. Regarding caring, a high level was already present at the beginning of the camp, which may have contributed to a ceiling effect. In addition, the context of the camp was more focused on enjoyment and a spirit of community rather than on individual achievement and success. Consequently, factors like personal effort, individual self-efficacy and problem-solving skills might not have been the primary focus, leading to stable values of these variables over time.

The remarkable increase in positive emotions combined with a stable low level of negative emotions indicates that, overall, positive experiences were made during the camp, even despite the simplicity of living in tents as well as dealing with fatigue and heavy rain during the second data collection. In addition, this finding is consistent with the literature as it confirms that positive emotions, compared to negative emotions, are more strongly influenced by daily experiences (Watson et al., 1988; Diener et al., 1999). Furthermore, participants’ increased readiness to contribute to the community shows that the Swiss Guide and Scout Movement, in line with the intention of PYD approaches, have a valuable impact on fostering not only the individual, but also the collective development (Lerner et al., 2019; Burkhard et al., 2020).

All significant changes were consistently explained by caring support received from group leaders. The higher the received caring support was, the more favorable the adolescents’ personal resources, positive emotions, self-esteem, and readiness to contribute to community developed during the camp. The role of a supportive environment became particularly important when dealing with participants who had below-average levels in personal resources, well-being, and readiness to contribute to the community at the beginning of the camp. Caring support from group leaders influenced whether these adolescents perceived an increase or a decrease in their development during the two weeks. Overall, supportive care from the leaders, and probably also their role model (Bonell et al., 2015; Holt et al., 2017), seemed to foster the most participants’ skills, attitudes and positive life expectations, and may have been a protective factor against the negative impacts of frustrations and difficulties in camp life (e.g., Chu et al., 2010).

As hypothesized, the effect of received caring support from group leaders on changes in positive emotions, self-esteem, and readiness to contribute to the community was partially to fully mediated by changes in personal resources. These findings confirm that a supportive environment has an influence on the affective and evaluative dimensions of well-being and the readiness to contribute to the community by enhancing individuals’ social and emotional skills and positive life expectations (Durlak et al., 2010; Ciocanel et al., 2017; Lerner et al., 2019). Furthermore, these findings also illustrate the complex interaction of multiple factors that are responsible for psychological and social development. In our findings, e.g., optimism, empathy, as well as assertiveness all together explained the positive changes in the readiness to take an active role in the community and to make a difference. Thus, having confidence in life and oneself, being oriented toward others as well as standing up for oneself altogether contribute not only to individual, but also to collective development (Lerner et al., 2019).

However, enhanced emotional self-control did not significantly relate to changes in well-being and readiness to contribute to the community. This may reflect the findings from literature that emotional self-control is mainly important for the regulation of negative social behavior, such as aggression and bullying (Skeen et al., 2019; Mertens et al., 2022), which was not the focus of this research.

Finally, it can be stated that the observed effects were rather small. However, considering the short duration of the camp and the rather high intraindividual stabilities in the most variables under examination, the findings are promising as they indicate that a large proportion of the participants benefited from the camp and from the caring support provided by their group leaders (Chu et al., 2010; Gariépy et al., 2016; Holt et al., 2017). Given the fact that the Swiss Guide and Scout Movement is a youth organization in which children and young people are active on most Saturdays and experience several camps, a cumulative effect is to be expected.

### 4.1 Strengths and limitations

The main strengths of this study are the pretest-posttest design, and the large sample size.

However, several limitations exist and should be kept in mind when interpreting the findings of this study. It was not a randomized controlled trial, meaning that the selection of participating Venture Scouts groups was not random, and a systematic bias cannot be excluded. Nevertheless, considering the minimal influences of family, school, or other leisure activities during the camp in a peripheral Swiss mountain valley, the observed changes in the various constructs can be largely attributed to Scouting activities.

There may be a self-selection bias in youth taking part in camps. Personal dispositions and already developed skills may have influenced the decision to attend the camp, as individuals tend to choose settings in which they can develop in accordance with the resources and competencies already gained (Diener and Fujita, 1995; Proctor et al., 2009). Familiarity with the camp’s life may also have influenced this decision, and, in addition, it may also have affected the likelihood of augmenting personal resources (see Gerber et al., 2022).

The results may also be limited because the camp was not a typical summer camp in terms of size, some program activities such as concerts, and the opportunities to meet peers from across the entire country and even from other countries. The focus in the camp was on maintaining the classic Scouting activities as described in the Swiss Guide and Scout Movement guidelines (Kalcsics et al., 2011). The study should be replicated in ordinary summer camps where individual groups and their leaders are responsible in designing meaningful activities and creating a supportive environment. An additional research question should focus on the participants’ perceived quality of the camp.

The camp took place under the Youth and Sports Program of the Swiss Federal Office of Sport. This means that each group was required to be physically active for four hours a day. Physical activity not only affects physical well-being, but also emotional and psychosocial well-being (Immordino-Yang et al., 2019). Thus, part of our results could also be explained by the high level of physical activity among camp participants. For this reason, physical activity and physical well-being should also be investigated in future studies.

There is also the question of reciprocal effects between the development of personal resources, well-being, and readiness to contribute to the community. In future studies, a third measurement point should be included to examine not only the extent to which changes in personal resources benefit well-being and the readiness to contribute to the community, but also how the latter conversely contribute to a safe place for learning and the development of personal resources. A third measurement point would also be necessary to investigate the sustainability of the positive developments beyond the camp.

Finally, the study focused on adolescents. It is important to examine the impact of camps on younger members, for example, in terms of the type of support that is particularly helpful and necessary for children, compared to adolescents (Chu et al., 2010; Holt et al., 2017; Kirchhoff and Keller, 2021). In addition, further research should examine whether young people benefit from camps in a similar way, regardless of their socio-economic or cultural background.

### 4.2 Conclusion

The National Jamboree of the Swiss Guide and Scout Movement required a collective effort. Every participant had to work together in solidarity to construct the new “home” for the upcoming two weeks and bring it to life. The camp offered the young people plenty of creative space to realize their own ideas and at the same time demanded joint decisions based on democratic principles.

By organizing camps in nature, youth organizations can make an important contribution to PYD. Such camps not only promote physical activity and provide enjoyment, but also help young people to develop practical and socio-emotional skills. In addition, camp activities contribute to a better well-being, and an increased motivation for community building.

In order to actively contribute to PYD, it is important for youth organizations to ensure the quality of support provided by well-trained group leaders. This can be achieved through the implementation of mentorship programs where experienced young adults act as leaders, guiding and supporting younger colleagues. This approach promotes a culture of continuous learning and skills development within the organization. Furthermore, youth organizations should not only focus on camps, but also on accompanying young people in their development over many years to form a strong community with long-lasting supportive relationships.

## Data availability statement

The datasets presented in this study can be found in online repositories. The names of the repository/repositories and accession number(s) can be found at: Open Science Framework, https://osf.io/t3x2j/.

## Ethics statement

The study was conducted in line with the ethical research guidelines as approved by the ethics committee of the Faculty of Arts and Social Sciences of the University of Zurich/Switzerland (approval number 22.6.12). In addition, the study was conducted in accordance with the local legislation and institutional requirements. The participants provided their written informed consent to participate in this study.

## Author contributions

EK: Conceptualization, Data curation, Formal analysis, Investigation, Methodology, Project administration, Visualization, Writing – original draft, Writing – review & editing. RK: Conceptualization, Investigation, Project administration, Resources, Supervision, Writing – review & editing, Methodology. BB: Conceptualization, Funding acquisition, Investigation, Project administration, Resources, Writing – review & editing.
