# Supplementary material for: Empowering young people—the impact of camp experiences on personal resources, well-being, and community building
Source: Front Psychol. 2024 Feb 14;15:1348050. doi: 10.3389/fpsyg.2024.1348050 (PMC10899333; doi:10.3389/fpsyg.2024.1348050)
Supplement: Supplementary file 1 [file Data_Sheet_1.PDF]

Supplementary Material

Empowering Young People – The Impact of Camp Experiences on Personal Resources, Well-Being, and Community Building

Esther Kirchhoff*, Roger Keller, Barbara Blanc

*** Correspondence:** Corresponding Author: esther.kirchhoff@phzh.ch

1 Supplementary Figures

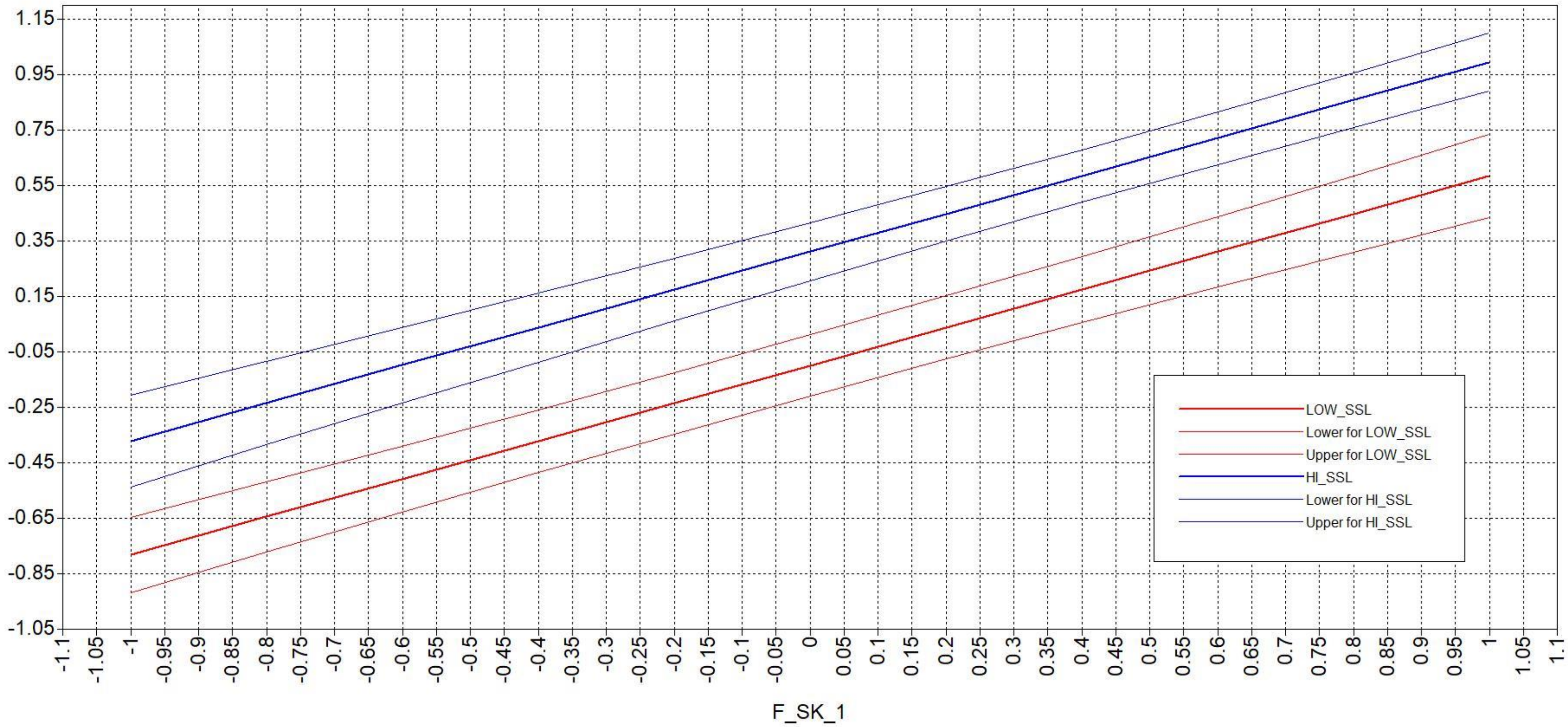

Figure S1. Graphical analysis of the moderation effect of caring support from group leaders on emotional self-control.

Legend: SSL = Caring support from group leaders. LOW_SSL = -1 *SD* value of caring support. HI_SSL = +1 *SD* value of caring support. F_SK_1 = emotional self-control.

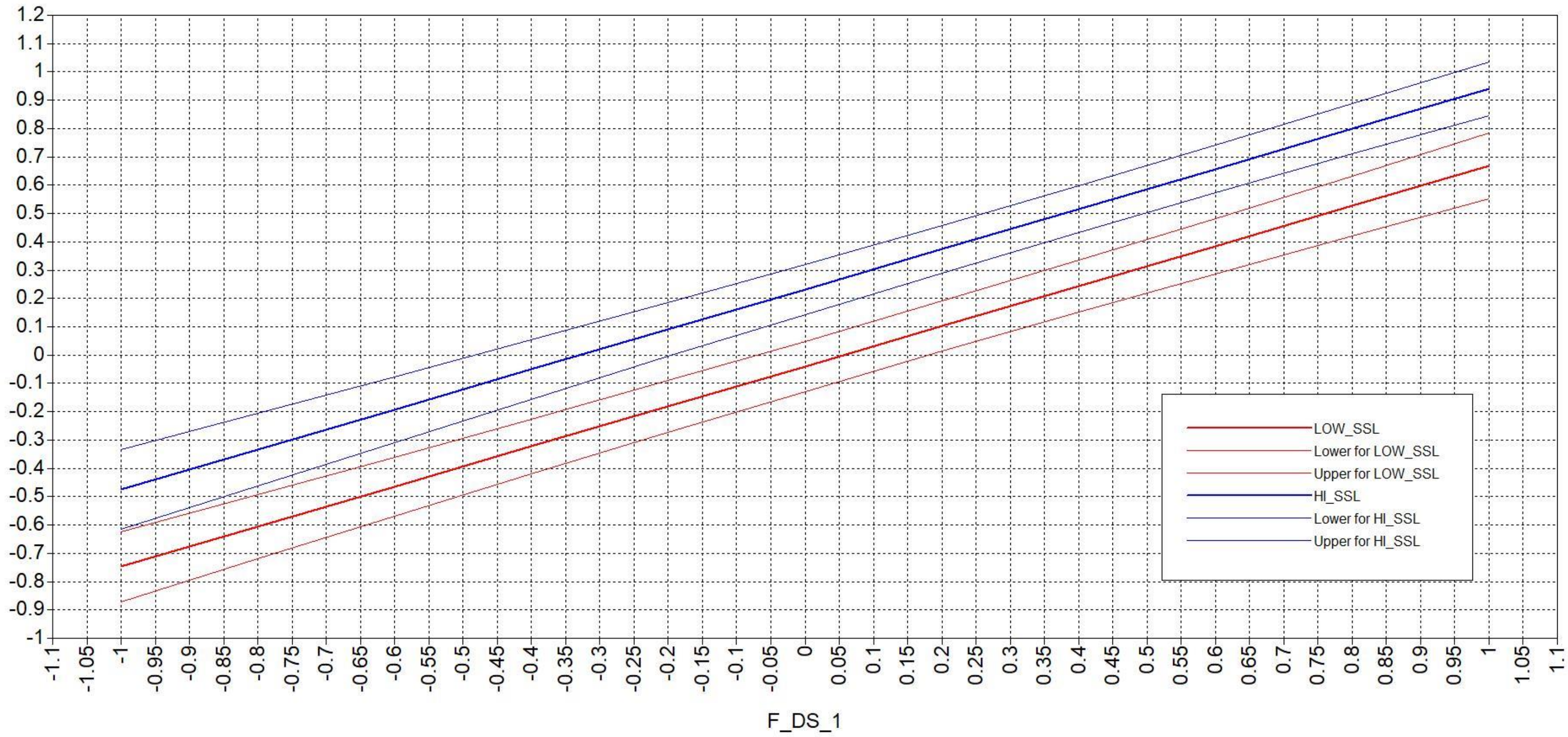

Figure S2. Graphical analysis of the moderation effect of caring support from group leaders on assertiveness.

Legend: SSL = Caring support from group leaders. LOW_SSL = -1 *SD* value of caring support. HI_SSL = +1 *SD* value of caring support. F_DS_1 = assertiveness.

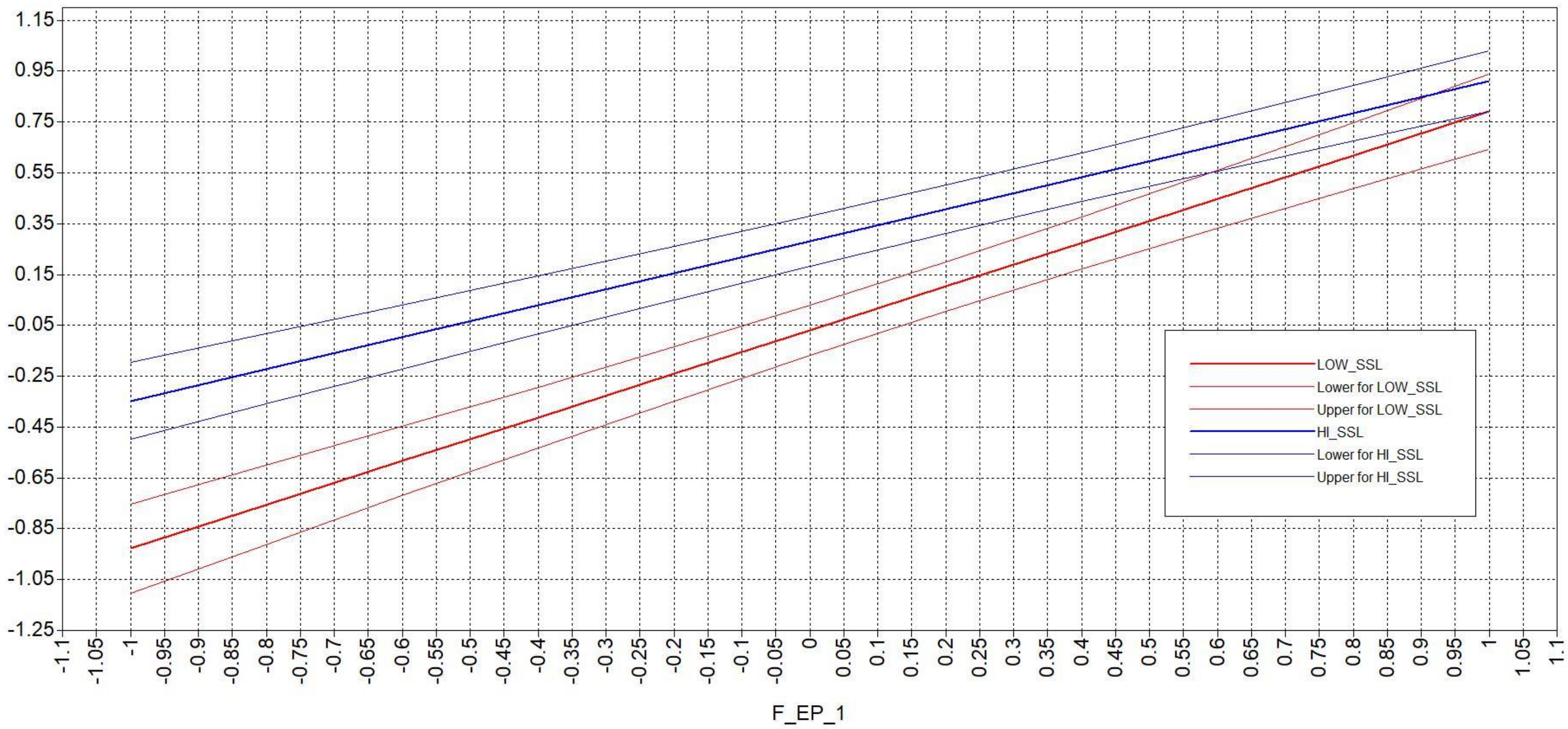

Figure S3. Graphical analysis of the moderation effect of caring support from group leaders on empathy.

Legend: SSL = Caring support from group leaders. LOW_SSL = -1 *SD* value of caring support. HI_SSL = +1 *SD* value of caring support. F_EP_1 = empathy / perspective-taking.

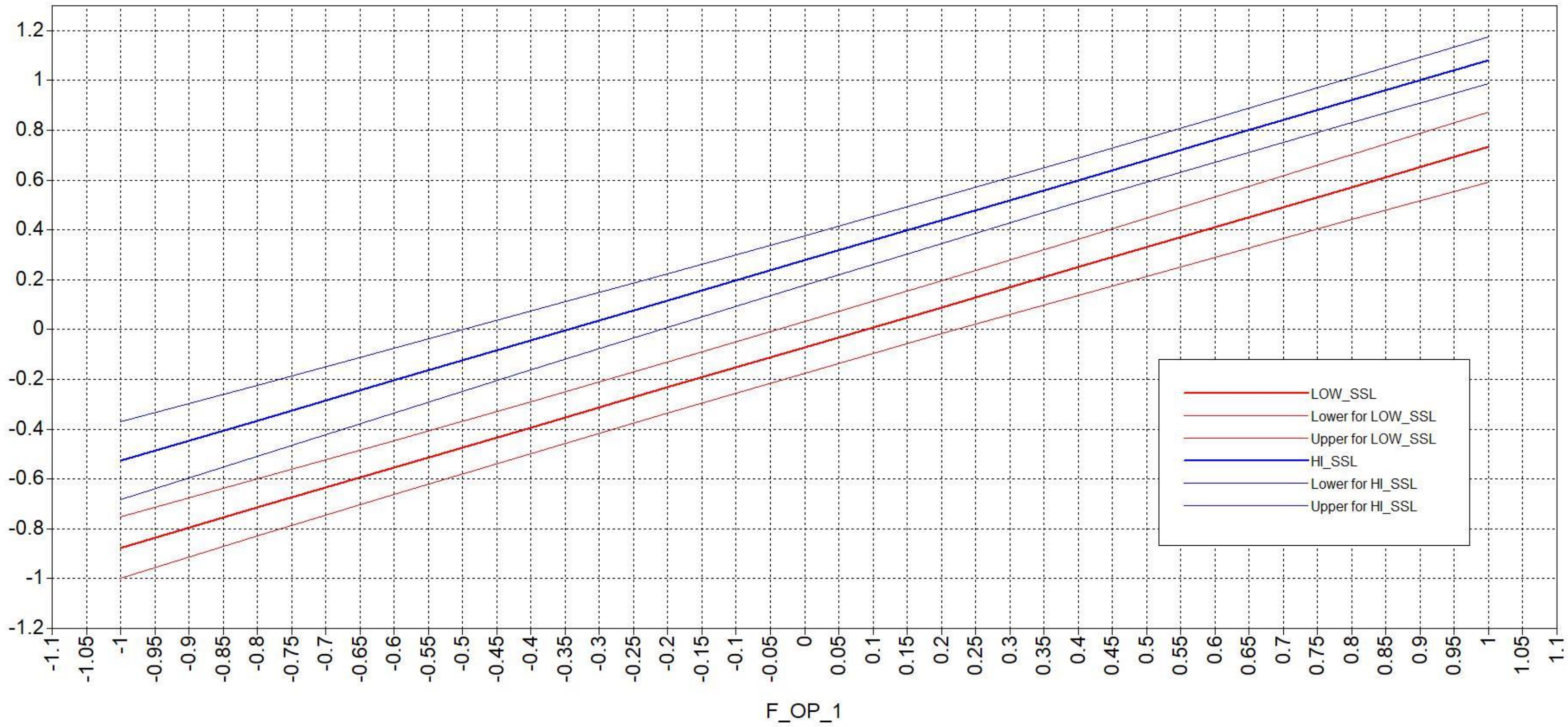

Figure S4. Graphical analysis of the moderation effect of caring support from group leaders on optimism.

Legend: SSL = Caring support from group leaders. LOW_SSL = -1 *SD* value of caring support. HI_SSL = +1 *SD* value of caring support. F_PP_1 = optimism.

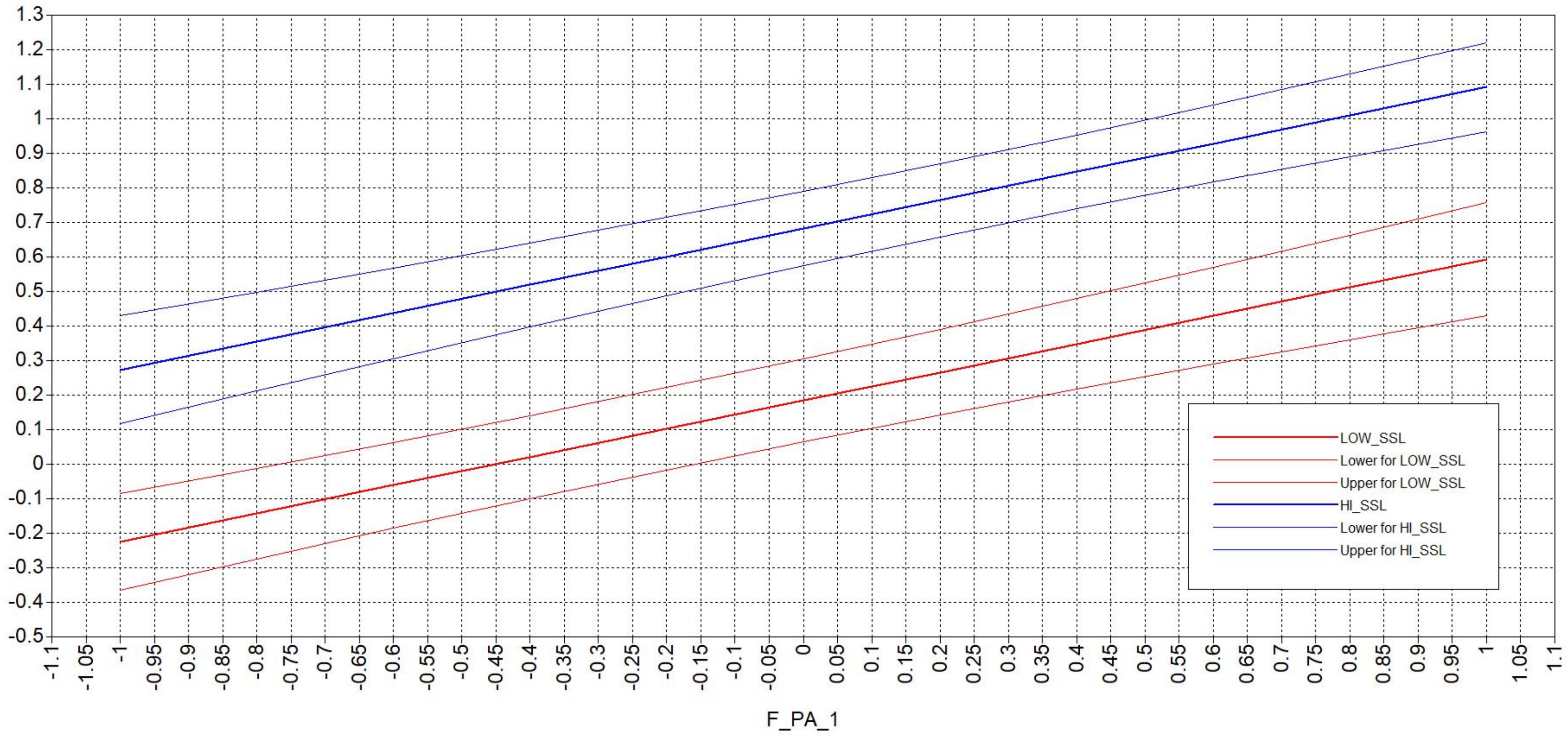

Figure S5. Graphical analysis of the moderation effect of caring support from group leaders on positive affect (PA).

Legend: SSL = Caring support from group leaders. LOW_SSL = -1 *SD* value of caring support. HI_SSL = +1 *SD* value of caring support. F_PA_1 = Positive affect.

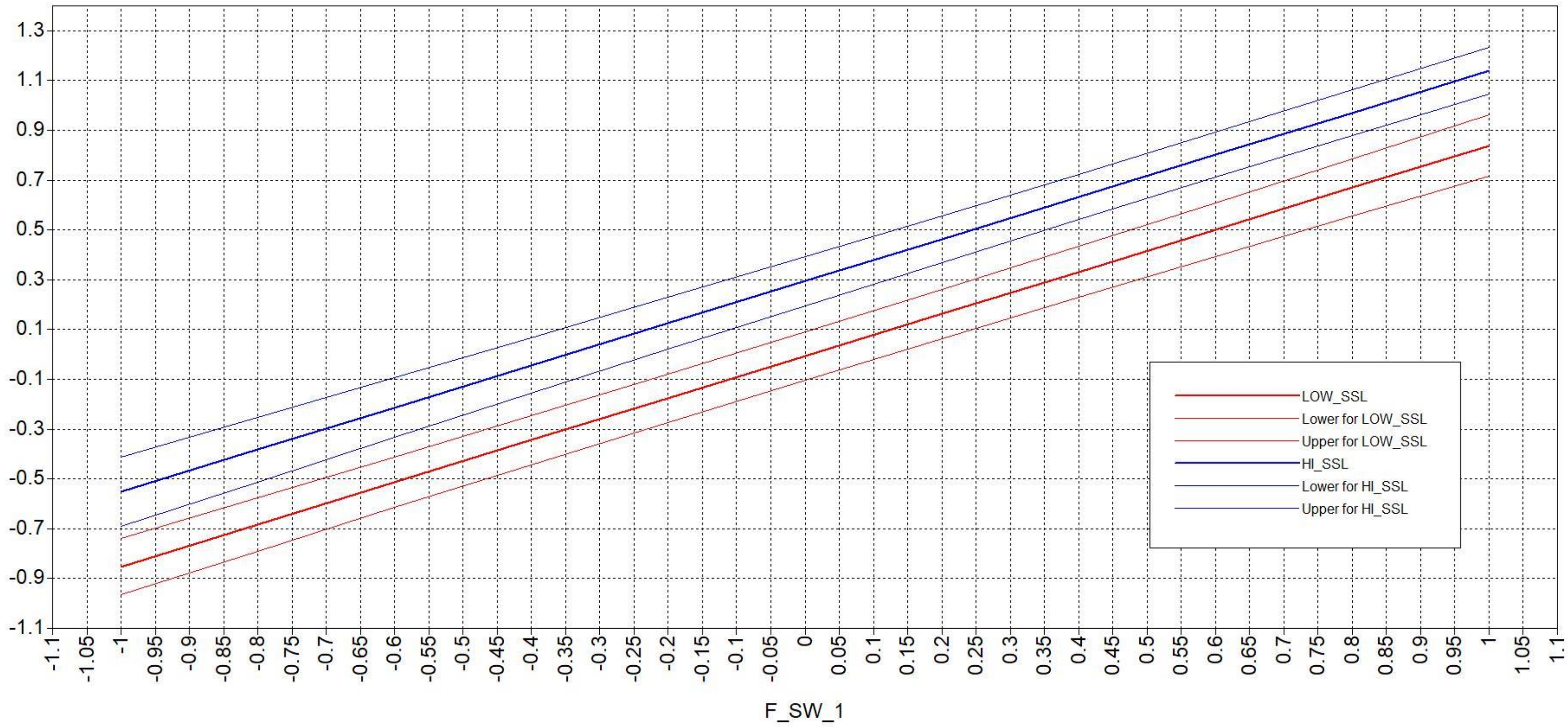

Figure S6. Graphical analysis of the moderation effect of caring support from group leaders on self-esteem.

Legend: SSL = Caring support from group leaders. LOW_SSL = -1 *SD* value of caring support. HI_SSL = +1 *SD* value of caring support. F_SW_1 = self-esteem.

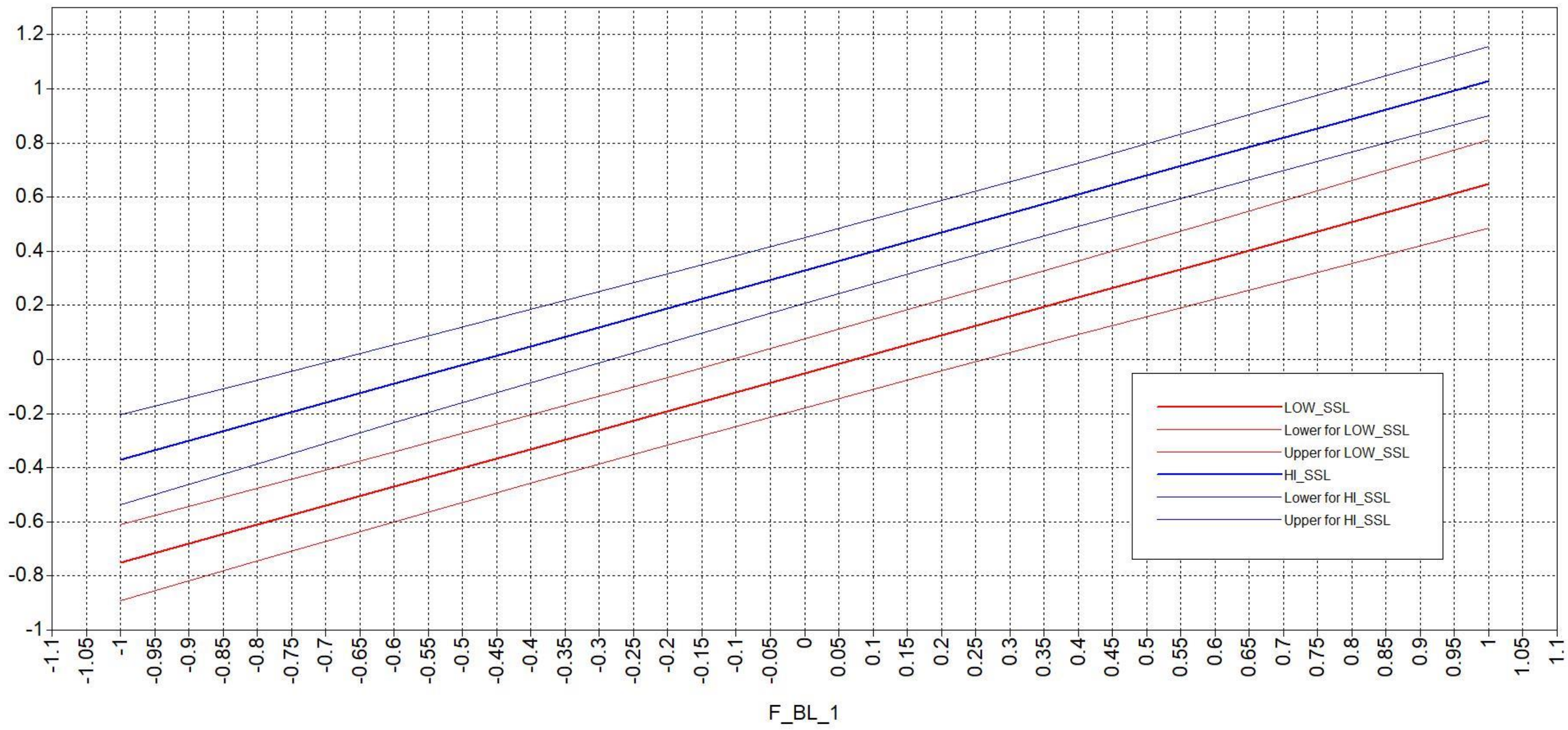

Figure S7. Graphical analysis of the moderation effect of caring support from group leaders on readiness to contribute to the community.

Legend: SSL = Caring support from group leaders. LOW_SSL = -1 *SD* value of caring support. HI_SSL = +1 *SD* value of caring support. F_BL_1 = readiness to contribute to the community.
